# Scoping Review on Epigenetic Mechanisms in Primary Immune Thrombocytopenia

**DOI:** 10.3390/genes14030555

**Published:** 2023-02-23

**Authors:** Jian Hong Tan, Ahmad Hazim Syakir Ahmad Azahari, Adli Ali, Noor Akmal Shareela Ismail

**Affiliations:** 1 Department of Paediatric, Faculty of Medicine, Universiti Kebangsaan Malaysia, Jalan Yaacob Latif, Cheras, Kuala Lumpur 56000, Malaysia; 2 Research Centre, Hospital Tunku Ampuan Besar Tuanku Aishah Rohani, UKM Specialist Children’s Hospital, Universiti Kebangsaan Malaysia, Jalan Yaacob Latif, Cheras, Kuala Lumpur 56000, Malaysia; 3 Department of Biochemistry, Faculty of Medicine, Universiti Kebangsaan Malaysia, Jalan Yaacob Latif, Cheras, Kuala Lumpur 56000, Malaysia

**Keywords:** autoimmune, epigenetic, blood disorder, DNA methylation, histone modification, lncRNA, miRNA

## Abstract

Immune Thrombocytopenia (ITP) is an autoimmune blood disorder that involves multiple pathways responsible for the homeostasis of the immune system. Numerous pieces of literature have proposed the potential of immune-related genes as diagnostic and prognostic biomarkers, which mostly implicate the role of B cells and T cells in the pathogenesis of ITP. However, a more in-depth understanding is required of how these immune-related genes are regulated. Thus, this scoping review aims to collate evidence and further elucidate each possible epigenetics mechanism in the regulation of immunological pathways pertinent to the pathogenesis of ITP. This encompasses DNA methylation, histone modification, and non-coding RNA. A total of 41 studies were scrutinized to further clarify how each of the epigenetics mechanisms is related to the pathogenesis of ITP. Identifying epigenetics mechanisms will provide a new paradigm that may assist in the diagnosis and treatment of immune thrombocytopenia.

## 1. Introduction

Immune Thrombocytopenia (ITP) is an autoimmune blood disorder that clinically manifests with an abnormally low platelet count of below 100,000 per microliter (100 × 109/L), which increases the risk of bleeding and bruising in affected individuals. The clinical features and manifestations that are common to ITP range from mild bleeding tendencies such as petechia, bruises and gum bleeding to the manifestation of severe bleeding, such as intracranial hemorrhage, gastrointestinal or genitourinary bleeding and severe menstrual bleeding [1]. ITP has three distinct phases, namely the initial phase (up to 3 months), the persistent phase (between 3 to 12 months), and the chronic phase (more than 12 months). Currently, there is no diagnostic test specifically for isolated ITP and this requires a series of diagnostic approaches to rule out other causes through detailed patient history, physical examination, and investigations. Treatment of ITP mainly includes platelet transfusions, glucocorticoids, intravenous immune globulin, splenectomy, and several other therapy options [2,3,4].

ITP can be classified into primary ITP and secondary ITP based on its etiologies, in which primary ITP refers to isolated thrombocytopenia with the absence of any other disorders or diseases [2,5,6,7]. Secondary ITP refers to all types of ITP excluding primary ITP, which is often caused by immunodeficiency and autoimmune diseases, and chronic infections. Examples of disorders causing secondary ITP include systemic lupus erythematosus (SLE), antiphospholipid syndrome (APLA), Helicobacter pylori infection, human immunodeficiency virus (HIV) infection, or hepatitis C virus (HCV) infection [8]. The incidence rate of ITP in adults is 1.6 to 3.9 per 100,000 person-years, while in children the incidence is between 1.1 to 5.8 per 100,000 person-years [9]. It is higher among females with female to male ratio ranging from 2.5:1 to 2.93:1 [10].

Immune thrombocytopenia (ITP) is a highly complex autoimmune condition, the cause of which is still being investigated and understood. The clinical features can be explained by a low platelet count due to increased antibody-mediated platelet destruction, or insufficient platelet production due to antibody-mediated inhibition of platelet production [8]. However, recent studies so far have found several other cell types that contribute to ITP pathogenesis including B cells, CD4+ T cells, CD8+ T cells (cytotoxic T-cell), macrophages, dendritic cells, regulatory T cells (Treg), and regulatory B cells (Breg) [11,12]. Classically, antiplatelet antibodies secreted by auto-reactive B lymphocytes are considered the main immunological abnormality in ITP. Autoreactive antibodies have been reportedly linked to dysfunctional T- and B- cells causing platelets to be destroyed, leading to abnormalities in thrombopoiesis and megakaryopoiesis [13,14]. In ITP, Treg cells can become deficient, resulting in dysfunctional regulation of the immune response towards platelets [15]. As such, several studies proposed that unbalanced Th17, Th0, and Th1 profiles may result in excessive autoimmune response, and subsequently induce a proinflammatory environment and contribute to platelets’ destruction [16,17]. Moreover, the presence of cytotoxic CD8+ T cells can directly destroy platelets and kill megakaryocytes which produce platelets [18,19].

Genetic predispositions could contribute to the development of ITP. In a microarray genetic-based study, clear discrimination between healthy individuals and individuals with ITP was observed, based on the gene expression profile [20]. Polymorphism is suggested to be a key player in the pathogenesis of ITP. A polymorphism in the CTLA4 gene, which is responsible for regulating the function of helper T cells leads to a higher susceptibility to ITP [21]. Another study on immune checkpoint-related single-nucleotide polymorphisms (SNPs) has elucidated several nucleotide changes in CD28, PD1, DNAM1, and LAG3 and associated them with the susceptibility and the therapeutic response to ITP [22].

Recent studies also have revealed several main epigenetic mechanisms and their role in the etiology of ITP which include DNA methylation, histone modification, and non-coding RNA (ncRNA) [23,24]. DNA methylation serves as a regulator of gene expression by recruiting proteins involved in gene repression or by inhibiting transcription factors from binding to DNA [25]. Non-coding RNA (ncRNA) plays an important role in many diverse biological pathways. The intervention of ncRNAs can interfere with gene expression, transcription, and translation processes [26,27,28,29]. Histone modifications are involved in altering the histone tails, thus playing significant roles in biological processes related to the regulation of chromatin and DNA expression. These histone modifications include acetylation, methylation, ubiquitylation, phosphorylation, sumoylation, ADP-ribosylation, and citrullination [30]. Recently, studies on the function of epigenetics in human diseases are expanding. However, the current literature on epigenetic mechanisms that are related to ITP is insufficient. Thus, this scoping review aims to provide an update and overview of epigenetic mechanisms that are associated with the pathogenesis of ITP.

## 2. Materials and Methods

### 2.1. Search Strategy and Selection Criteria

The methodology for this study was based on the guidelines provided by Preferred Reporting Items for Systematic Reviews and Meta-Analyses (PRISMA). The search was conducted in five electronic databases: PubMed, Scopus, Web of Science, Ovid and Science Direct. Search queries were based on the name of the disease and its epigenetic mechanisms: “Immune Thrombocytopenia” OR “Idiopathic Thrombocytopenic Purpura” OR “Immune Thrombocytopenic Purpura” with AND “DNA methylation”, AND “Histone modification”, AND “lncRNA”, AND “miRNA”. The last search was done on 30th December 2022.

Articles were eligible for inclusion if they described the primary immune thrombocytopenia with relevant epigenetic mechanisms to the disease. Studies on the use of drugs to treat epigenetic mechanisms, pregnancy care for patients with ITP, secondary immune thrombocytopenia, or without the epigenetic mechanisms were excluded from this review. Papers available with abstracts only were also excluded. Only articles published in English were retrieved. Both adult and pediatric groups were included in this review. Epigenetic mechanisms and pathways involved only in primary immune thrombocytopenia identified through experimental research are the outcomes of interest for this scoping review.

### 2.2. Data Collection

The literature search was performed and collated by two independent reviewers (TJH and AHSAA). The discrepancies in determining the inclusivity of the articles were discussed and the decision made by the content experts (AA and NASI). All citations were imported into the desktop reference management application, Mendeley Reference Manager. Duplicate citations were initially identified and removed manually, together with duplicates that were found later in the process. The titles, abstracts, and keywords of each citation were assessed for relevance to the study before being exported into an Excel file with the citation records. Justifications for omitting citations were recorded. Full articles that met the requirements of inclusion were retrieved for further review. The following details were recorded on the Excel data extraction form: authors, study titles, publication years, source titles, volumes, issues, and digital object identifiers (DOI). The Joanna Briggs Institute (JBI) critical appraisal tools were used to evaluate the quality of the included studies.

## 3. Results

**Figure 1:**
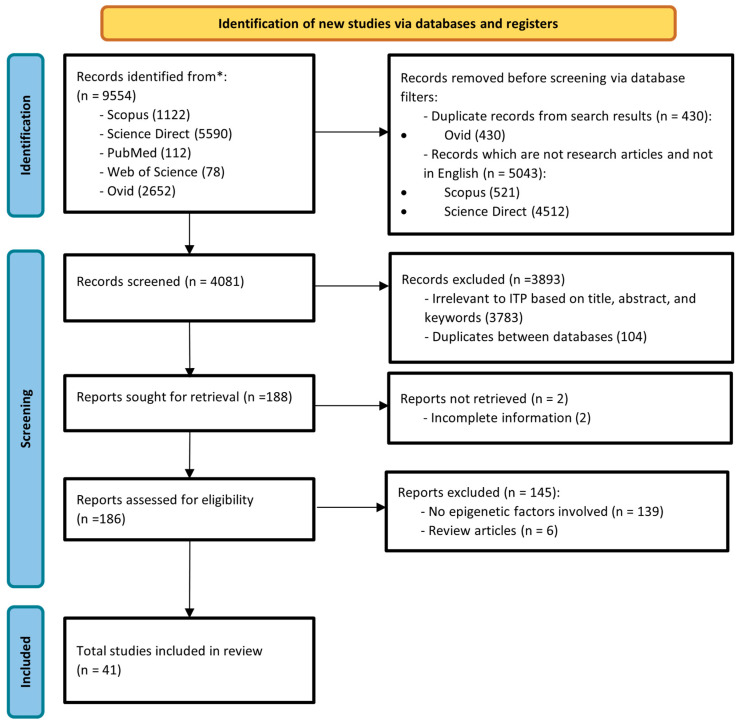
Flowchart of record identification related to epigenetic mechanisms in immune thrombocytopenia purpura (ITP). * databases of Scopus, Science Direct, PubMed, Web of Science and Ovid.

The initial search results yielded 9554 records from all five electronic databases, consisting of articles, books, case reports, and drug studies. Through the filter function of respective databases, duplicates and records that are not research articles or not in English, were removed before screening. The remaining 4081 were screened solely based on title, abstract, and keywords related to ITP. Duplicates between databases were also removed. A total of 3783 records were deemed irrelevant to ITP and 104 records were further removed as duplicates between databases. Following this, 188 records were retrieved for the full text except for 2 records. Out of these 186 records, 41 articles were included in this review. The remaining excluded records consist of drug treatment studies, books, case reports, and studies on ITP but not related to epigenetic mechanisms (Figure 1).

### 3.1. DNA Methylation

DNA methylation is one of the epigenetic factors that silence a specific gene expression. Seven studies revealed DNA methylation of different genes to be associated with ITP. Aberrant methylation, such as hypermethylation and hypomethylation can disrupt normal functional gene expression and cell function, and in the case of ITP, it could disrupt T-cell function [15] (Table 1). Several studies on ITP have demonstrated abnormalities in DNA methylation. Foxp3 promoter [31] and NOTCH1 [32] are found to be hypermethylated in ITP. When NOTCH1 is hypermethylated, the expression of Th1-cell-related.

Cytokines is downregulated, whereas Th2 cell-related cytokines’ expression were upregulated [32]. A significantly greater rate of methylation was found on CpG sites of the perforin and IFN-γ gene promoter, but a correlation was unable to be established between ITP and methylation on CpG sites of both genes [33]. CD70 gene promoter was found hypomethylated, thus CD70 was overexpressed. In turn, the expression of methyltransferase genes, DNMT1, DNMT3A and DNMT3B was significantly higher in ITP [34]. On the contrary, two studies found DNMT3A and DNMT3B mRNA expressions were significantly lower, suggesting that the DNMT3A gene is highly methylated [35,36]. Plasma levels of S-adenosylhomocysteine (SAH) were significantly elevated in acute, and chronic ITP was significantly elevated which suggests a close relationship with DNMT3A [35,36]. One study found that patients with ITP have significantly reduced global DNA methylation accompanied by a notably lower level with the expression of methylated DNA binding domain protein 2 and 4 (MBD2 and MBD4) mRNA levels in ITP [37].

**Table 1:** Studies on epigenetic mechanism in immune thrombocytopenia purpura (ITP).

Epigenetic Mechanisms	Type of Study	Methodology	Major Findings	Pathway Affected/Functions	References
**DNA Methylation**					
FOXP3 hypermethylation	In vitro and in silico	PBMCs extraction, DNA extraction, DNA methylation analysis (MALDI-TOF, MassCLEAVE)	The Foxp3 promoter has CpG sites with higher levels of methylation. The CpG-6 promoter of Foxp3 is methylated at various amounts in different ITP patients.	Maintenance and function of Treg cells	[31]
CD70 (TNFSF7) promoter hypomethylation	In vitro	PBMCs and CD4+ cells isolation, flow cytometric analysis, DNA extraction and bisulfate modification, methylation-sensitive high-resolution melting analysis, RNA isolation, quantitative real-time PCR	Overexpression of CD70, DNMTs and MBD2. Hypomethylation of CD70 promoter in CD4+ T cells of ITP patients. CD70 transcription levels negatively correlated with methylation indices but positively correlated with DNMT1, DNMT3A, DNMT3B.	CD70-CD27 costimulatory pathway, CD28-CD80/86 pathway, ERK signal pathway	[34]
DNMT3A mRNA expression decreased as a result of aberrant DNA methylation	In vitro	PBMCs extraction, RNA isolation, reverse transcription, quantitative real-time PCR, reverse-phase HPLC	Reduced DNMT3A mRNA expression, and increased SAH levels.	Not mentioned	[35]
Increased methylation on promoter of perforin and IFN-γ but not associated with ITP	In vitro	RNA isolation, reverse transcription, and quantitative real-time PCR; DNA extraction and bisulfate sequencing	Expression of IFN-γ, IL-4, Foxp3, and perforin is negatively correlated with methylation of respective promoters	Th1 polarization response	[33]
Decreased MBD2 and MBD4 gene expression, hypomethylation of genome	In vitro	PBMCs and CD4+ cells isolation, RNA and DNA extraction, ELISA for dmC, quantitative real-time PCR	Reduced global DNA methylation in CD4^+^ cells, reduced MBD2 and MBD4 mRNA expression, methylation index negatively correlated with MBD2 and MBD4 expression.	Global methylation and demethylation activity	[37]
Hypermethylated NOTCH1 in Th1 and Th2 cell differentiation pathways	In vitro	PBMCs and bone marrow mononuclear cells (BMMCs) isolation, reduced representation bisulfite sequencing (RRBS), BSMAP software, wANNOVAR, GO analysis, KEGG analysis, Luminex assay, quantitative real-time PCR, and ELISA	NOTCH1 and TYK2	Th1 and Th2 pathways	[32]
Decreased DNA methyltransferase 3A and 3B mRNA expression in peripheral blood mononuclear cells and increased plasma SAH concentration		PBMC, isolation of total RNA, reverse transcription, real-time quantitative PCR, reversed phase HPLC	3A and 3B mRNA	SAH pathway	[36]
**Histone modification**					
Global hypomethylation of H3K9 in CD4^+^ T cells	In vitro	RNA isolation, reverse transcription, real-time PCR, global histone H3K4/H3K9 methylation assay	Global histone H3K9 hypomethylation with reduced expression level of EZH2 and SUV39H2, while SUV39H1 has no changes.	H3K9 trimethylation and H3K27 methylation	[38]
Increased citrullinated histone and histone-DNA complexes.	In vitro	Plasma extraction, specific ELISA bioassays for S100A8/A9, histone/DNA complexes, citrullinated histone H3, cfDNA, ADAMTS13, and Anti-ADAMTS13 IgG	Plasma inflammatory mediators, NETosis markers, cfDNA play a role in pathogenesis of ITP	NETosis and inflammatory process	[39]
**LncRNA**					
Decreased lncRNA PVT1 expression in ITP patients	In vitro	CD4+ T cell isolation, Th17 cell differentiation, Flow cytometry, quantitative real-time PCR, T cell transfection, ELISA, Western blot, ubiquitination analysis	In ITP patients, PVT1 expression was downregulated whereas Th17 cell expression was elevated. PVT1 overexpression reduced IL-17, RORt, and NOTCH1 levels as well as the quantity of Th17 cells.	Notch signaling pathway	[40]
Eight lncRNAs and eleven miRNAs associated with ITP	In silico	Affymetrix array, GO analysis, KEGG analysis, miRNA prediction: (starBase), Protein-protein and coexpression network mapping (Cytoscape)	7 genes, 8 lncRNA, 11 miRNAs are found to be associated with ITP pathogenesis	Around 30 pathways related to autoimmunity	[41]
Increased MALAT1 and THRIL expression	In vitro	RNA extraction from blood, reverse transcription	MALAT1 and THRIL were upregulated, and positive correlation found between the expression level of lncRNA.	TLR2 signaling pathway; Alternative splicing regulation	[42]
Aberrant lncRNA of ENST00000440492, ENST00000528366, NR_038920, and ENST00000552576 expression	In vitro and in silico	RNA isolation from blood, microarray, KEGG Pathway analysis, GO analysis, quantitative real-time PCR, CNC analysis	A total of 4 immune-related lncRNAs were found associated with genes and proteins related to autoimmune disease	TNF signaling pathway, granulocyte macrophage colony-stimulating factor production, coreceptor activity, cytokine–cytokine receptor interaction, chemokine signaling pathway	[43]
Decreased TMEVPG1 expression	In vitro	Plasma extraction, peripheral blood mononuclear cell culture, real-time PCR, ELISA for cytokine	TMEVPG1 may increase IFN-γ transcription, and IFN-γ overexpression negative feedback controlled by TMEVPG1 expression	IFN-γ mediated signaling pathway	[44]
GAS5 regulates Th17 and STAT3 ubiquitination	In vitro and in vivo	PBMC extraction, CD4+ T cell transfection and induction of Th17 differentiation, quantitative real-time PCR, flow cytometry, Western blot analysis, ELISA, RNA pull-down assay, RNA binding protein immunoprecipitation (RIP), ubiquitination assay	In both humans and mice with ITP, GAS5 expression was downregulated. GAS5 regulates differentiation of Th17 through TRAF6-mediated ubiquitination of STAT3	MAPK pathway and Th17 differentiation	[45]
MEG3 regulates miR-125a-5p, CXCL3, and ratio of Treg/Th17	In vitro	CD4+ T cell extraction, flow cytometry analysis, RNA isolation, real-time PCR, Western blot analysis, ELISA, RNA pull-down assay, luciferase reporter gene assay	Elevated MEG3 expression in ITP patients. Treg/Th17 imbalance brought on by miR-125a-5p. MEG3 inhibits miR-125a-5p.	Th17/Treg balance ratio	[46]
The Ifng antisense RNA 1 (IFNG-AS1) and growth arrest-specific transcript 5 (GAS5)	In vitro	RNA extraction from venous blood, reverse transcription, quantitative real-time PCR (qPCR) for detection of long non-coding RNAs	lncRNAs IFNG-AS1 and GAS5 is overexpressed in childhood ITP.	Not mentioned	[47]
**MicroRNA**					
MicroRNA increased gene expression of CXCL13 and IL-21 in patients with ITP.	In vitro and in silico	T cell extraction from blood, RNA extraction, miRNA labeling and hybridization, DNA microarray, GO analysis (TargetScan, Miranda, Cytoscape, ClueGO).	Identified 17 microRNAs that are linked to the expression of 57 immune system-related target genes.	T-cell activation, regulation of immunoglobulin production	[48]
Increased miR-302c-3p, miR-483-5p, miR-223-3p, and miR-597 expression. Decreased miR-544a and miR-302a-3p expression.	In vitro	miRNA isolation from plasma with quantitative real-time PCR.	Found 7 miRNA as biomarkers for further research of ITP pathogenesis.	Not Mentioned	[49]
miR-98-5p downregulates IGF2BP1 and upregulates p53	In vitro and in vivo	Human mesenchymal stem cells extraction, murine model with ITP, miRNA microarray, Western blot analysis, and ELISA Assay.	MSC apoptosis is correlated with miR-98-5p overexpression. MiR-98-5p downregulates IGF2BP1 and upregulates p53.	MSC apoptosis; IGF-2/PI3K/Akt Pathway	[50]
Aberrant expression of microRNA in CD4 + cells contributes to Th17/Treg imbalance	In vivo	Cohort of 52 ITP patients and 56 healthy controls. RT-PCR, flowcytometry, Western blot analysis.	The expression of miRNAs related to helper T or Treg cells was found to regulate the Th17/Treg ratio in CD4+ T cells. Lower levels of miR-99a were observed in ITP patients compared with healthy controls, while higher levels of miR-182-5p and miR183–5p were seen among ITP patients. Positive correlation between increased percentage of Treg and decreased levels of miRNA-99a was also noted in ITP patients.	Th17/Treg pathway	[51]
Bone marrow mesenchymal stem cell-derived exosomes induce the Th17/Treg imbalance in immune thrombocytopenia through miR-146a-5p/IRAK1 axis.	In vitro and In vivo	Density-gradient centrifugation to separate BMSCs, culturing the separated BMSC, identification of BMSCs by culturing them up to third generation.	BMSCs-exosomes’ treatment significantly reduced the Th17/Treg ratio in CD4 + T cells	Th17/Treg pathway	[52]
Differential expression of miR-106b-5p and miR-200c-3p in newly diagnosed versus chronic ITP patients.	In vivo and in silico	Microarray, RT-PCR, bioinformatic analysis.	Three specific microRNA molecules -miR-106b–5p, miR200c–3p and mir92a-3p showed significantly different expressions among all groups studied. MiR 106b 5 p and 200 c-3p had higher levels of expression in patients with ITP compared to normal controls; and chronic ITP vs. newly diagnosed cases.	Not mentioned	[53]
Downregulation of microRNA-155-5p prevents immune thrombocytopenia.	In vitro	Transfection of macrophages and PBMC’s with treated plasmids, ELISA.	miR-155-5p was upregulated and SOCS1 down regulated in PBMCs and macrophages from ITP. Inhibition of miR-155-5p or upregulation of SOCS1 facilitated the M2 polarization, increased M2/M1 ratio. Silencing SOCS1 blocks PD1/PDL1 pathway and upregulates miR-155-5p.	PDL/PDI pathway through Socs1	[54]
miR-21 and miR-150 expression.		RT-PCR.	A significant relationship between the expression of miR-21 with hemoglobin, hematocrit and red blood cell hemoglobin concentration, but not miR-150.	Not mentioned	[55]
Increased miR-155 expression correlated with serum cytokine profiles.	In vitro	RT-PCR, ELISA.	Increased plasma IL-17A and decreased IL-4, IL-10 and TGF-β1 levels in ITP patients. miR-155 levels were negatively correlated with platelet counts, SOCS1 mRNA levels, and the plasma levels of IL-4, IL-10 and TGF-β1, but positively correlated with plasma IL-17A levels.	SOCS1 pathway	[56]
Integrated mRNA and miRNA with deregulation of the cellular stress response in bone marrow mesenchymal stem cells derived ITP.	In vitro	Bone marrow samples of ITP patients taken, isolation expansion and characterization of MSC’s, RNA extraction, microarray study.	A total of 740 genes and 32 miRNAs were differentially expressed between ITP patients and controls. A compromised unfolded protein response (UPR) and decreased DNA transcription were shown to be significantly related to MSC-ITP.	Not mentioned	[57]
MicroRNA expression profile in Treg cells in ITP.	In vitro	Platelet collection of ITP patients, miRNA microarray analysis.	miR-155–5p, miR-146b–5p, and miR-142–3p significantly decreased in Tregs from patients with ITP.	Tregs pathway	[58]
MicroRNA profiling of platelets from immune thrombocytopenia and target gene prediction.	In vitro	Platelet collection of ITP patients and healthy controls, RT-qPCR, microarray analysis.	A total of 115 miRNAs are differentially expressed in ITP patients compared to healthy controls. There were 6 specific miRNA targets which may be involved in processes such as apoptosis (cell death involving programmed cell self-destruction) and adhesion related to the pathogenesis of ITP.	Not mentioned	[59]
MicroRNA-21-5p regulates CD3+ T lymphocytes through VCL and LTF.	In silico	Bioinformatic analysis.	S100A8 regulates CD3+ T lymphocytes in ITP patients. MiR-21-5p regulates the differentially expressed gene LTF by inhibiting the core downstream target gene VCL and participates in the immune mechanism of T lymphocytes in ITP patients. miR-155-5p involved in the immunoregulatory mechanism of T lymphocytes in ITP patients.	Not mentioned	[60]
miR-106b-5p induces immune imbalance of Treg/Th17 in immune thrombocytopenic purpura through NR4A3/Foxp3 pathway.	In vitro and in vivo	miRNA expression levels, cell proliferation rates, cytokine production profiles, gene sequencing, qRT-PCR, Western blot, ELISA.	miR-106b-5p was elevated in peripheral blood of patients with ITP, and NR4A3 expression was decreased. sh-NR4A3 significantly decreased Foxp3 and TGF-β expressions, indicating that NR4A3 may regulate Treg differentiation via Foxp3. NR4A3 was identified as a target of miR-106b-5p, and miR-106b-5p was able to negatively modulate NR4A3 expression. miR-106b-5p induced immune imbalance of Treg/Th17 through NR4A3. Silencing miR-106b-5p promoted Treg differentiation and increased the number of platelets.	NR4A3/Foxp3 pathway	[61]
MiRNA-148b-3p targeting SOCS3 inhibits macrophage M2 polarization by JAK2/STAT3 pathway.	In vitro and in vivo	Platelet collection of ITP patients, real-Time PCR, Western blot, dual-luciferase reporter gene assay	A significant correlation between miR-148b-3p expression and platelet count. Suppression of miR-148b-3p or up-regulation of SOCS3 promoted macrophage M2 polarization by inhibiting JAK2/STAT3 pathway.	JAK2/STAT3 pathway	[62]
Correlation between different expression of miRNA levels between the ITP patients and healthy children.	In vitro	Platelet collection of ITP patients, qRT-PCR.	Seven miRNAs (miR-302c-3p, miR-483-5p, miR-410, miRNA 302a 3P, miRNA 223 3P and miRNA 597) had significantly different expression levels between the ITP patients and healthy children.	Not mentioned	[63]
Characterization of miRNA in ITP patients.	In vitro	Plasma collection of ITP patients, microRNA microarray analysis, qRT-PCR.	Upregulated miRNAs (miR-320c, miR-642b-3p, miR-1275, miR-3141, miR-4270, miR-4499, miR-4739 and miR-6126) and three down-regulated miRNAs (miR-144-3p, miR-1281 and miR-3162-3p) in ITP patients.	Not mentioned	[64]
miRNA through exosome trafficking to the cell membrane.	In vitro	Plasma collection of ITP patients, exosome extraction, qRT-PCR, RNA sequencing, Western blot.	Three differentially expressed miRNAs (miR-584-5p, miR-142-5p and miR-29b-3p) were identified in ITP patients.	Not mentioned	[65]
Reduced expression of miR409-3p.	In vitro	Total RNA extraction, DNA synthesis, RT PCR assays.	MIR409-3p expression was decreased in PBMCs of active ITP patients but recovered after effective therapy.	Not mentioned	[66]
Reduced miR130A is involved in ITP via targeting TGFB1 and IL18.	In vitro	Peripheral blood collected, miRNA array and TaqMan real-time polymerase chain reaction.	MiR130A expression significantly decreased in PBMCs of ITP patients. MiR130A targeted the TGFB1 and IL18 genes. Post-treatment upregulates expression of miR130A and TGFB1, downregulates IL18 expression.	TGFB1 pathway via IL18	[67]
The aberrant expression of microRNAs and correlations with T cell subsets.	In vitro	Peripheral blood collected, miRNA array and TaqMan real-time polymerase chain reaction.	miR-146a positively correlated with the frequencies of Treg cells and platelet counts. miR-146a expression upregulation contributed to the differentiation of Th17 and Treg in ITP patients. miR-146a may be involved in Tregs differentiation and function.	Th17/Treg pathway	[68]
Increased let-7b-5p is associated with enhanced BAFF-R expression and B cell survival in immune thrombocytopenia.	In vitro	B cells extraction, RNA extraction and quantitative real-time PCR, Western Blot, flow cytometry.	Overexpression of let-7b-5p in B cells enhanced the expression of surface BAFF-R and promoted B cell survival. let-7b-5p enhanced the phosphorylation of NF-κB2 p100 and upregulated the expression of survival factor Bcl-xL after BAFF induction.	NF-κB2 pathway	[69]
The increased expression of miR-146 predicts poor prognosis of ITP.	In vitro	Plasma and megakaryocytes extraction, RNA extraction, (qRT-PCR).	miR-146 is overexpressed in ITP patients. Higher expression of miR-146 is associated with poorer prognosis, lower platelet count, and increased risk of relapse.	Not mentioned	[70]
miR-557 inhibits the differentiation and maturation of megakaryocytes.	In vitro and in vivo	MTT assay, CFSE staining, qRT-PCR.	miR-557 inhibitor increased the numbers of platelets and megakaryocytes and improved the symptoms of ITP. miR-557 inhibitor regulates apoptosis-related genes: Caspase-3 and Bax inhibition, and upregulation of bcl-2, p-Akt and p-ERK.	Akt/ERK pathway	[71]

### 3.2. Histone Modification

Histone modification leads to either chromatin compaction or decompaction, which may cause silencing effects on certain genes. Two histone modification mechanisms were highlighted in two in vitro studies associated with ITP. A significant association of global histone H3K9 hypomethylation was found in CD4+ T cells of patients with active ITP. Expression of SUV39H2 and EZH2 were downregulated in CD4+ T cells of patients with active ITP when compared to the patients in remission and healthy controls [38]. Plasma levels of S100A8/A9, histone/DNA complexes, citrullinated histone H3 (CitH3), and cell-free DNA (cfDNA) were found to increase significantly in ITP patients, but significantly lowered in remission. Correlation analyses indicate that increased CitH3 is positively correlated with organ damage, and significantly correlated with the mortality of ITP patients [39].

### 3.3. LncRNA

Long noncoding RNA (lncRNA) is a transcript sequence that consists of 200 nucleotides and often binds to the DNA sequence to inhibit specific gene transcription. It serves as a multi-function gene transcription regulator, by modifying transcription activity and post-transcriptional regulation [32,36,38]. MALAT1 [42], THRIL [42], MEG3 [46], IFNG-AS1, and GAS5 genes [47] related to inflammation processes were upregulated in ITP. MALAT1 and THRIL established a substantial positive association between the expression levels of MALAT1, THRIL and the white blood cell count [42]. When CD4+ T cells are treated with dexamethasone, it significantly reduced the level of MEG3 expression and increased the expression of miR-125a-5p in peripheral CD4+ T cells. Foxp3 expression is promoted while RORγt expression is inhibited by MEG3 downregulation and miR-125a-5p overexpression [46]. IFNG-AS1 and GAS5 were found to be overexpressed in childhood ITP, persistent ITP, and chronic ITP, especially when compared with patients who completely responded to treatment. Furthermore, both expressions of IFNG-AS1 and GAS5 are significantly negatively correlated with platelet count after therapy [47].

PVT1, TMEVPG1 and GAS5 are also found to be downregulated in ITP [40,44,45]. PVT1 overexpression causes increased ubiquitination of NOTCH1, reducing the amount of Th17 cells, and levels of IL-17, RORγt, and NOTCH1 [40]. TMEVPG1 expression shows a significantly positive correlation with IFN-γ mRNA expression and platelet counts in ITP patients. It is suggested that TMEVPG1 increases IFN-γ transcription, but when IFN-γ is overexpressed, it causes negative feedback that decreases TMEVPG1 expression [44]. Low levels of lncRNA GAS5 and a higher level of STAT3 were observed in PBMCs of ITP patients, resulting in a higher percentage of Th17 cells and a lower percentage of Treg cells [45]. When GAS5 is overexpressed, expression of RORγt and IL-17 were in turn decreased, with no effect on Foxp3 and IL-10 expression. Increased ubiquitination of STAT3 immunoprecipitated by TRAF6 was observed after overexpression of GAS5 [45]. Reversal of GAS5-induced degradation of STAT3 has been observed by MG132 (a proteasome inhibitor) administration. When naïve CD4+ T cells were treated with CHX (protein synthesis inhibitor) for GAS5 overexpression, the STAT3 level was improved [45].

Two in-silico studies discovered ten lncRNAs importantly related to the pathogenesis of autoimmune diseases and immune functions [41,43]. NR_038920 and ENST00000528366 were both associated with proteins involved in the pathogenesis of autoimmune diseases [43]. Another eight lncRNAs: LOC101927237, LINC00515, LOC101927066, LOC440028, RP11-161D15.1, LOC101929312, AX747630, and LOC100506406 are involved in several pathways and/or functions: positive regulation of inflammatory response, cellular response to cGMP, ephrin receptor signaling pathway, chronic inflammatory response, forelimb morphogenesis, stem cell population maintenance, cell junction assembly, positive regulation of cell growth, chemical synaptic transmission, and inflammatory response [41].

### 3.4. MicroRNA

Small non-coding RNAs known as microRNAs control the expression of genes by post-transcriptional degradation or translational repression. A total of 24 studies highlighted the role of the miRNA mechanism in ITP. Significantly increased levels of CXCL13 and IL-21 were observed in the plasma of individuals with ITP. These two biomarkers are involved in the differentiation of plasma cells and B-memory lymphocytes [48]. A pro-apoptotic role of miR-98-5p was identified in mesenchymal stem cells (MSCs) of ITP, where the miR-98-5p targets IGF2BP1, where knockdown of IGF2BP1 increased levels of apoptosis in healthy MSCs. MiR-98-5p directly targets and downregulates IGF-2 through post-transcriptional repression and upregulates p53 [50]. MiR-146a-5p is highly expressed by bone marrow mesenchymal stem cells’ (BMSCs) exosomes which directly inhibit interleukin-1 receptor-associated kinase 1 (IRAK1) expression in CD4+ T cells. However, IRAK1 overexpression increased Th17 cells and decreased Tregs. MiR-146a-5p regulates the imbalance of Th17/Treg in CD4 + T cells through IRAK1 inhibition [52]. The expression of miR-155-5p was found to be upregulated and downregulated haploinsufficiency of suppressor of cytokine signaling 1 (SOCS1) gene in PBMCs and macrophages. Inhibition of miR-155-5p or upregulation of SOCS1 facilitated the M2 polarization, resulting in an increased ratio of M2/M1. Inhibition of SOCS1 promoted the progression of ITP by blocking the PDL/PDI pathway, while upregulation of miR-155-5P increases platelet abundance and downregulates expression of SOCS1 [54]. Increased miR-155 in ITP is positively correlated with IL-17A and negatively correlated with platelet counts, SOCS1 mRNA levels, and plasma levels of IL-4, IL-10 and TGF-β1. It is suspected that miR-155 regulates cytokine profiles via targeting SOCS1 [56] S100A8, miR-21-5p, and miR-155-5p increased in CD3+T lymphocytes of ITP. MiR-21-5p regulates CD3+T Lymphocytes inhibiting VCL and LTF [60].

MiR-106b-5p was elevated in the peripheral blood of patients with ITP, and intracellular transcription factor, nuclear receptor 4A3 (NR4A3) expression was decreased. miR-106b-5p targets NR4A3 which is suspected to downregulate Treg differentiation via Foxp3, inducing immune imbalance of Treg/Th17. Inhibition of miR-106b-5p promoted Treg differentiation and increased the number of platelets [61]. MiR-148b-3p was upregulated in ITP and significantly correlated with platelet count. Suppression of miR-148b-3p or upregulation of SOCS3 promoted macrophage M2 polarization by inhibiting the JAK2/STAT3 pathway [62]. Increased let-7b-5p in B cells was elevated in ITP, which enhances the expression of surface BAFF Receptor (BAFFR) and phosphorylation of NF-κB2 p100, which in turn promotes increased levels of anti-apoptotic factor, Bcl-xL for B cell survival [69]. Increased expression of miR-146 correlated with poor prognosis of ITP, as higher expression of miR-146 is associated with lower platelet count and increased risk of disease relapse [70]. In ITP, miR-4689, miR-557, miR197-3p, and miR-3945 were significantly upregulated, and the expressions of miR-634 and miR-144-3p in immune platelets were significantly reduced. Thrombopoietin and miR-557 inhibitors were able to alleviate negative effects, including increasing the numbers of platelets and megakaryocytes [71].

A combination of lower levels of miR-99a, higher levels of miR-182-5p and miR-183–5p were seen among ITP patients. A positive correlation between the increased percentage of Treg cells and decreased levels of miR-99a was also noted in ITP patients. A negative correlation between platelet count and expression of miR-183–5p in CD4+ cells from severe ITP patients was observed [51]. MiR409-3p expression was decreased in PBMCs of active ITP patients but recovered after effective therapy [66]. MiR130A expression was significantly decreased in PBMCs of ITP patients. Reduced miR130A is involved in ITP via targeting TGFB1 and IL18. The expression level of miR130A in PBMCs from ITP patients was significantly lower than normal controls. MiR130A also targeted TGFB1 and IL-18. Post-ITP treatment upregulated the expression of miR130A and TGFB1 whereas IL18 expression was downregulated [67]. Expression of miR-146a, miR-326 or miR-142-3p in ITP was lower. The frequencies of Treg cells were decreased, whereas the frequencies of Th17 and Th22 cells were increased significantly in ITP patients compared to those in controls. The expression levels of miR-142-3p and miR-146a were negatively correlated with Th17 cells. The expression of miR-146a was positively correlated with the frequencies of Treg cells and platelet counts. MiR-146a expression upregulated with agomir and dexamethasone contributed to the increase in miR-146a and differentiation of Th17 and Treg [68].

Several differential expression and correlation studies were also identified. Between ITP patients and healthy children, seven miRNAs: miR-302c-3p, miR-483-5p, miR-410, miR-302a-3P, miRNA-223-3P and miRNA-597 had significantly different expression levels [63]. Another study found 23 miRNAs had different levels between ITP patients and healthy controls: miR-320c, miR-642b-3p, miR-1275, miR-3141, miR-4270, miR-4499, miR-4739, miR-6126, miR-144-3p, miR-1281 and miR-3162-3p [64]. MicroRNAs associated with platelet apoptosis and adhesion in ITP were: hsa-miR-548a-5p, hsa-miR-1185-2-3p, hsa-miR-30a-3p, hsa-miR-6867-5p, hsa-miR-765 and hsa-miR-3125 [59]. A total of 14 miRNAs were identified that expressed differently between newly diagnosed and chronic ITP. Among them, miR-106b-5-p and miR200c–3p had higher levels of expression in patients with ITP compared to normal controls [53]. MiR-21 is correlated with hemoglobin, hematocrit, and red blood cell concentration [55]. Unfolded protein response (UPR) and decreased DNA transcription are significantly related to mesenchymal stem cells [57]. Three differentially expressed miRNAs; miR-584-5p, miR-142-5p and miR-29b-3p are related to plasma-derived exosomes and were identified in ITP patients [65]. In Tregs from patients with ITP: miR-155–5p, miR-146b–5p, and miR-142–3p were significantly decreased compared with healthy control [58]. 

## 4. Discussion

In nearly all human diseases and disorders, genetics plays a role with varying degrees of influence. However, a growing amount of research has also pointed out that epigenetics plays an important role in the development of diseases. DNA methylation, histone modification, non-coding RNA (ncRNA) and miRNA interventions are the major mechanisms [22,23]. Epigenetics is now established as playing a significant function in organ development, the manifestation of human disease, our interaction with the environment, and human evolution [72,73,74,75].

In this review, nearly all studies found epigenetic mechanisms involving T cells. Only one study involved miRNA that affects the B-cells [69]. Thrombocytopenia has various causes including drug-induced thrombocytopenia (DITP), blood cancers, cancer treatments, and infections [76], thus, making primary ITP a diagnosis of exclusion. The pathogenesis of DITP is largely dependent on the structure of the drug substance and its function [77]. DITP is associated with several mechanisms which include the drug molecules interacting with drug-dependent antibodies, platelet surface proteins, and glycoprotein IIb/IIIa [78,79]. However, there is a lack of studies on epigenetics in DITP. Viral infections such as dengue virus, Human Immunodeficiency Virus (HIV) and SARS Coronavirus 2 (COVID-19) have been found to cause thrombocytopenia [80]. There is limited literature on the epigenetics of thrombocytopenia caused by a viral infection, however, a recent longitudinal study found that severe fever with thrombocytopenia syndrome Virus (SFTSV) infection negatively affects modification and subsequently affects the transcriptome of the patients [81]. In vaccine-induced thrombotic thrombocytopenic purpura (VITT), citrullinated histone H3 significantly increased in regards to increasing NETosis [82].

Epigenetic mechanisms are important in the functions of apoptosis and inflammation in autoimmune diseases. Thus, it is beneficial for us to understand if any of the pathways found are unique to ITP. Unfortunately, the pathways found in this review are not unique to ITP, as the pathways found in ITP are also involved in various autoimmune diseases.

### 4.1. Pathogenesis of ITP

**Figure 2:**
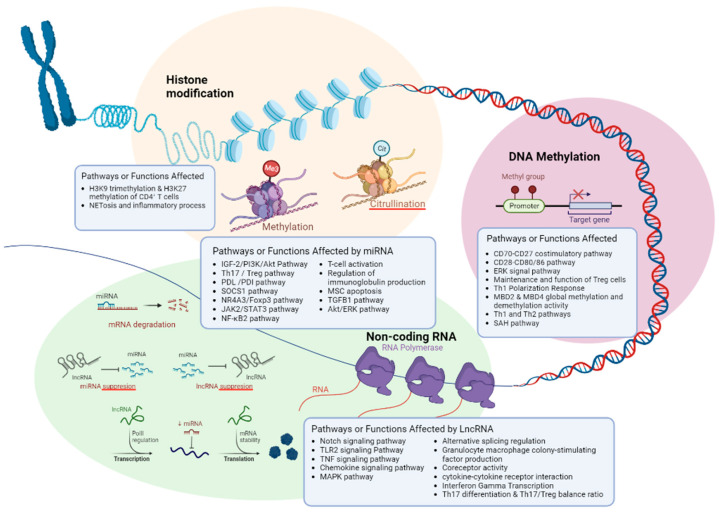
Epigenetic mechanisms in ITP. Created with BioRender.com (accessed on 1 February 2023).

The pathogenesis of ITP is a complex process that involves several immune cells, such as B cells, plasma cells, Tregs, Bregs, and cytotoxic CD8+ T cells (Figure 2). This process is closely tied to the destruction of platelets [83] and the deficiencies in megakaryopoiesis and thrombopoiesis [84,85]. The regulation of B cells and plasma cells is disrupted in ITP, leading to the aberrant production of autoantibodies that attack platelets and megakaryocytes. This results in their impairment and degradation in the spleen and liver [86,87]. Moreover, the cellular immune response is impacted, causing a reduction in Treg and Breg levels, contributing to an imbalance in Th CD4+ T cell subsets. This leads to the survival of autoreactive plasma cells and the formation of more autoantibodies. Cytotoxic CD8+ T lymphocytes are also activated in ITP, causing dysregulation of bone marrow homeostasis, and inducing platelet and megakaryocyte apoptosis [14]. T helper cells play a crucial role in the development of autoimmune responses. The major T cell subsets involved in ITP include T-helper 1 (Th1), T-helper 2 (Th2), T-helper 3 (Th3), T-helper 17 (Th17), and regulatory T cells (Tregs) [88]. Th1 and Th2 cells are responsible for driving the type-1 and type-2 pathways, respectively, which lead to cellular and humoral immunity [89]. Tregs, which make up 5-10% of the peripheral CD4+ T cell population, are critical for maintaining self-tolerance by suppressing immune responses. Th1, Th2, Th17, and Treg are among the T cells that are crucial for the development of ITP [11,14,17]. In ITP, the levels of Tregs are significantly reduced, leading to a skewed ratio of Tregs to Th17 cells in favor of Th17 cells, which promotes inflammation [90,91,92,93]. This could serve as an avenue for new prognostic biomarkers, potentially the NLRP3 inflammasome that correlates with release of the pro-inflammatory cytokines IL-1β, IL-18 and HGMB1 [94].

### 4.2. Th1/Th2 and Th17/Treg Imbalance

The significance of the ratio lies in the dynamic balances of Th1/Th2 and Treg/Th17 which are essential for keeping a healthy immune system. It has been demonstrated that disruption of the Treg/Th17 balance contributes to the pathogenesis of autoimmune disorders, including ITP [11,95,96]. Abnormal cytokine profiles including interferon-γ (IFN-γ), interleukin-2 (IL-2), interleukin-4 (IL-4), interleukin-10 (IL-10), interleukin-17 (IL-17), and transforming growth factor β (TGF-β) reflect the levels of Th1/Th2 and Treg/Th17. Thus, cytokine profiles can indicate if one is healthy or under a pathogenic condition [97,98]. In another study, natural killer (NK) cells (CD16+56+), interleukin- 6 (IL-6), and tumor necrosis factor α (TNFα) were also included as indicators of ITP [99]. It was found that cytokine polymorphism and insufficient cytokine secretion can increase the susceptibility and severity of ITP [100,101]. The imbalance of Th1/Th2 and Th17/Treg in ITP patients may be caused by an imbalance of plasmacytoid dendritic cells (pDC)/myeloid dendritic cells (mDC), as well as an increase in IL-6, IL-12, and IL-23. These factors also cause an increase in the differentiation of CD4+ T cells into Th1 and Th17 cells [99]. This could be the key factor contributing to the imbalance between Th17/Treg and Th1/Th2 in ITP patients [95,102,103]. The imbalance of Th1/Th2 and Th17/Treg ratio involves several pathways. The Notch signaling pathway has been found to play a critical role in a variety of biological processes [104,105] including regulating the fate of T cell and B cells in autoimmune and lymphoproliferative diseases [106,107]. NOTCH1, NOTCH2, NOTCH3, NOTCH4, and Notch ligands are essential to this pathway [96]. Studies have shown that a lack of Notch-1 and Notch-2 leads to impaired CD4+ and CD8+ T cell function and increased susceptibility to infections. In particular, the Notch pathway has a bipotential switch that regulates the differentiation of T cells into Tregs and Th17 cells, and higher Notch1 expression levels have been observed in patients with ITP [108,109,110]. In SLE and psoriasis vulgaris (PV), low levels of PVT1 have been linked to immune system dysfunction [111,112].

**Figure 3:**
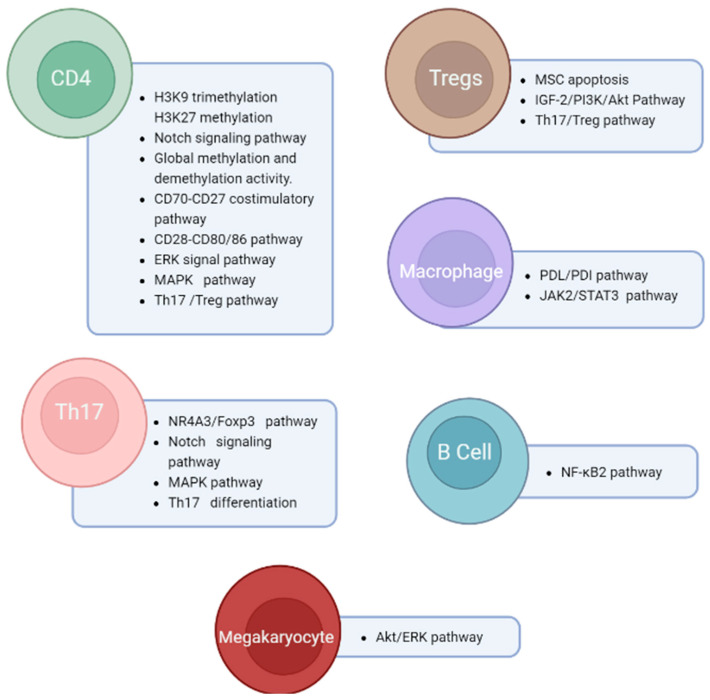
Pathways affected in ITP. Created with BioRender.com (accessed on 1 February 2023).

IFN-γ is a crucial cytokine to immunity against intracellular pathogens and tumor control [113]. TMEVPG1 has been reported to regulate Th1 cells through IFN-γ [114,115]. In Sjögren syndrome, another immune system disorder, upregulation of TMEVPG1 enhances Th1 cell response, and the knockdown of TMEVPG1 decreases the proportion of Th1 cells [116]. Imbalance of Th1 and Th2, where both Th1 dominance and Th2 dominance are known to cause and/or be involved with autoimmune dysfunctions [117]. Th1 is known for the development of autoimmune diseases via continuous activation of macrophage and T cells [118]. Involved in the innate immune response to pathogens, overactivation of the TLR2 signaling pathway results in autoimmune and inflammatory diseases. Similar to TLR2, other Toll-Like Receptors (TLR) such as TLR4 and TLR7 aberrant expression results in an overactive inflammatory response [119]. The PI3K/Akt pathway plays a role in regulatory T cell development, including the differentiation and function of Treg cells. The functional PI3K/Akt pathway is essential for functional and stable Treg cells, where aberrations in the PI3K/Akt pathway have resulted in immune disease [120,121,122,123]. MiR-98-5p is known for its regulation of cancer via proliferation and metastasis, where deregulation of miR-98-5p increases the proliferation of cancer and tumor cells [124,125]. Several studies have shown that miR-98-5p induces cell apoptosis via targeting components of functional pathways such as targeting MAP4K4 in the MAPK/ERK signaling pathway [125,126]. In ITP, mesenchymal stem cells (MSCs) are reported to be defective and show enhanced apoptosis [49], which results from miR-98-5p targeting insulin-like growth factor 2 mRNA binding protein 1 (IGF2BP1) which is a crucial mediator in the IGF-2/PI3K/Akt pathway (Figure 3).

### 4.3. Potential Epi-Biomarkers of ITP

DNA methylation is the additional methyl groups at cytosine phosphodiester-linked guanine (CpG) islands. In ITP patients, it was found that there is a high methylation level at CpG sites of the forkhead box P3 (FOXP3) promoter, resulting in lower FOXP3 gene expression which functions as a key player in the development and function of regulatory T cells [30]. FOXP3 is a protein involved in immune system responses which supposedly helps control the activity of various genes that are involved in regulating the immune system, especially regulatory T (Treg) cells [30].

The CD70-CD27 costimulatory pathway and CD28-CD80/86 pathway both play important roles in T cell activation and immune response [127,128], which has been observed in other autoimmune diseases [129]. The SAH pathway which is involved in methylation processes, including DNA methylation, RNA modification, and protein methylation, has been observed in cardiovascular disease [130]. Histone citrullination at H3 is known to be associated with neutrophil extracellular traps (NETs) and the NETosis process, which have been proposed as a biomarker for thrombosis [131,132]. NETS are involved in pro-inflammatory responses and pathogenesis of autoinflammatory and autoimmune diseases including SLE, RA, and Type 1 diabetes mellitus (T1DM) [133,134,135,136,137,138]. NETosis can promote thrombosis and thrombogenicity [139,140]. This could explain why CitH3 was elevated in ITP as thrombosis is one of the common symptoms of ITP. This also means the CitH3 can potentially be used as a biomarker for levels of thrombosis in ITP patients.

CD70 is a methylation susceptible immune gene from the tumor necrosis factor (TNF) family and is expressed on B cells, T cells and dendritic cells [104,105]. CD70 is overexpressed in SLE, SCLE, SSc, and primary Sjögren’s syndrome (pSS), because of demethylation of its promoter regulatory regions in CD4+ T cells [106]. Although the function of CD70 in Treg cells is not entirely understood, research has shown that its expression in expanded Tregs is linked to a severe loss of regulatory function [98]. MBD2 overexpression may be one of the factors contributing to the pathogenesis of ITP. Nonetheless, two separate studies reported otherwise, showing that the low expression of DNMT3A, DNMT3B, MBD2 and MBD4 in ITP and the hypomethylation of MBD2 and MBD4 may play an equally important role in the progression of ITP [36]. MBD2 can serve as both a transcriptional repressor and an enzyme with demethylase activity [141,142,143]. However, up to this date, no study compares the low and high methylation levels of MBD2 side by side concerning ITP. The true function of MBD2 in ITP should therefore be investigated. Studies involving MBD4, CD4+ T cells from SLE patients had considerably lower levels of MBD4 expression, which led to the overexpression and hypomethylation of the CD70 gene in these cells [144].

H3K9 methylation is known to be a gene-repressive function that mediates the silencing of invasive and repetitive sequences by preventing the expression of aberrant gene products and the activation of transposition and is associated with a condensed heterochromatin state [145,146,147]. Hypomethylation in this region can result in marked genomic instability [148]. H3K9 and H3K27 aberrant methylation was reported with aberrant expression of EZH2 and SUV39H2 and were specifically reported in SLE, SS, and RA [149]. Several pieces of literature have found that instead of SUV39H2, SUV39H1 was involved in the trimethylation of H3K9 [150,151,152]. This is because SUV39H1 and SUV39H2 have the same function but have different expression patterns. SUV39H1 shows little tissue preference except in the thymus, while SUV39H2 is more highly expressed in the brain, testis, and thymus [153]. Based on these studies, changes in histone modification were found to be significantly correlated with the pathogenesis of ITP, however, this area still required further study to identify the exact role of histone modification in the pathogenesis of ITP. Based on this review, a list of potential biomarkers were identified and are listed in Table 2.

### 4.4. Potential Treatment of ITP

There are several treatment options for ITP, and the choices of treatments depend on the severity and age of the patient [154]. Current therapies and management such as corticosteroids, IVIG, anti-D immunoglobulin, and dexamethasone have limitations, and some patients have reported having negative impacts on health-related quality of life [155]. A before-and-after treatment study done with thrombopoietin-receptor agonists (TPO-RA) (romiplostim and eltrombopag) has identified that miR-199a-5p and miR-221-3p can predict platelet response to TPO-RA-treatment before treatment [156]. Recent research has investigated decitabine as a treatment for ITP [157,158]. Decitabine is a demethylating agent that in low doses is reported to have three functions: (i) promote megakaryocyte maturation and platelet production through demethylating hypermethylated promoters of tumor necrosis factor-related apoptosis-inducing ligand (TRAIL) gene [159]; (ii) restores expression of programmed cell death protein 1 (PD-1), by demethylating hypermethylated PD-1 promoter in CD8+ T cells, which inhibits cytotoxic T lymphocytes (CTLs)-mediated platelet destruction [160] and (iii) enhances Tregs and restores the balance of T helper cell subtypes through inhibition of STAT3 activation which decreases STAT3 and AKT phosphorylation [160]. Low-dose decitabine demonstrated long-term efficacy in platelet production promotion and increasing Treg regulatory function [157]. Interestingly, there are three traditional Chinese medicinal drugs reported to treat ITP. QFRG has been reported to increase miR-181a expression and decrease TLR-4 expression in mice with ITP [161]. YQSX is reported to possibly interact with the MAPK signaling pathway and PI3K-AKT signaling pathway [162]. QSBLE is a Mongolian medicine reported to recover platelet count with minimal side effects. Collectively, both studies identified miRNA and proteins that were differentially expressed but were not able to explain the mechanism of QSBLE in ITP [163,164].

## 5. Conclusions

Currently, multiple studies have nominated several genetic and epigenetic biomarkers as important indicators for the diagnosis of ITP and its severity. However, a comprehensive understanding of how the interplay between these genes and epigenetic factors is required to further elucidate the pathogenesis and progression of ITP. Identification of the role of DNA methylation, histone modification, and non-coding RNA should be thoroughly researched, especially the specific mechanisms and their regulation to discover the latest, specific diagnostic biomarkers and subsequently assist with the management of ITP.

## Figures and Tables

**Table 2:** Potential Epi-biomarkers for Immune Thrombocytopenia Purpura (ITP).

Potential Epi-Biomarker	Known Related Functions	Reference(s)
CitH3	NETosis	[37]
MALAT1, THRIL	TLR2 signaling Pathway	[42]
CXCL13, IL-21	Plasma cells and B-memory lymphocytes	[48]
miR409-3p, miR-146, miR-106b-5p, miR-302c-3p, miR-483-5p, miR-223-3p, miR-597, miR-544a, miR-302a-3p, miR-410, miR-320c, miR-642b-3p, miR-1275, miR-3141, miR-4270, miR-4499, miR-4739, miR-6126, miR-144-3p, miR-1281 and miR-3162-3p, miR-106b-5-p, miR200c–3p, NR_038920 and ENST00000528366.	Immune-related	[43,49,53,61,63,64,66,70]
hsa-miR-548a-5p, hsa-miR-1185-2-3p, hsa-miR-30a-3p, hsa-miR-6867-5p, hsa-miR-765 and hsa-miR-3125	Platelet apoptosis and adhesion	[59]
miR-584-5p, miR-142-5p and miR-29b-3p	Plasma-derived exosomes	[65]
miR-155–5p, miR-146b–5p, and miR-142–3p CD70 H3K9 DNMT3A, DNMT3B, MBD2 and MBD4	Tregs Oxidative stress Histone modification Gene regulation	[58] [34] [38] [34,35]

## Data Availability

Not applicable.
